# A nano-predator of pathological MDMX construct by clearable supramolecular gold(I)-thiol-peptide complexes achieves safe and potent anti-tumor activity

**DOI:** 10.7150/thno.59020

**Published:** 2021-05-03

**Authors:** Siqi Yan, Jin Yan, Dan Liu, Xiang Li, Qianyan Kang, Weiming You, Jinghua Zhang, Lei Wang, Zhiqi Tian, Wuyuan Lu, Wenjia Liu, Wangxiao He

**Affiliations:** 1 Department of Talent Highland, The First Affiliated Hospital of Xi'an Jiaotong University, Xi'an 710061, China.; 2 Ophthalmology Department, The First Affiliated Hospital of Xi'an Jiaotong University, Xi'an 710061, China.; 3 National & Local Joint Engineering Research Center of Biodiagnosis and Biotherapy, The Second Affiliated Hospital of Xi'an Jiaotong University, Xi'an 710004, China.; 4 School of Pharmacy, Second Military Medical University, Shanghai 200433, China.; 5 School of Public Health, Xi'an Jiaotong University Health Science Center, Xi'an 710061, China.; 6 Institute for Stem Cell & Regenerative Medicine, The Second Affiliated Hospital of Xi'an Jiaotong University, Xi'an 710004, China.; 7 Department of Cancer Biology, University of Cincinnati College of Medicine, Cincinnati, 45267 OH, USA.; 8 School of Basic Medicine, Fudan University, Shanghai 20433, China.

**Keywords:** Protein targeted degradation, Peptide, p53, Anti-cancer therapy, Supermolecule

## Abstract

As alternatives to small-molecular proteolysis-targeting chimeras (PROTAC), peptide-based molecular glues (MG) are a broad range of dual-functional ligands that simultaneously bind with targetable proteins and E3 ligases by mimicking proteinprotein interaction (PPI) partners.

**Methods:** Herein, we design a peptide-derived MG to target a tumor-driving protein, MDMX, for degradation, and nanoengineered it into a supramolecular gold(I)-thiol-peptide complex (Nano-MP) to implement the proteolysis recalcitrance, cellular internalization, and glutathione-triggered release. To optimize the tumor targeting, a pH-responsive macromolecule termed polyacryl sulfydryl imidazole (PSI) was synthesized to coat Nano-MP.

**Results:** As expected, Nano-MP@PSI induced the MDMX degradation by ubiquitination and subsequently restored the anti-cancer function of p53 and p73. Nano-MP@PSI revealed potent anti-cancer activities in an orthotopic xenograft mouse model of retinoblastoma by intraocular injection and a patient-derived xenograft model of malignant pancreatic cancer by systemic injection, while maintaining a favorable safety profile and showing a highly favorable clearable profile of excretion from the living body.

**Conclusion:** Collectively, this work not only provided a clinically viable paradigm for the treatment of a wide variety of tumors by multiple administration types, but, more importantly, it bridged the chasm between peptides and PROTACs, and likely reinvigorated the development of peptide-derived proteolysis-targeting chimeras for a great variety of diseases.

## Introduction

Nowadays, worldwide drug regulators have approved therapeutics against about 400 human proteins, and nearly all of them can be categorized as enzymes, transporters, and receptors 1. Despite the increasing number of identified therapeutic targets in the human proteome, more than 85% of that 3000 disease-causing proteins that fall into the categories of nonenzymatic proteins, transcription factors, and scaffolding protein, were considered to be “undruggable,” because these proteins cannot be targeted for the development of small molecule compounds to inhibit their biological function 1, 2. Even among the 13% of druggable targets (400 out of 3000), conventional therapeutic strategies on the occupation of active sites tend to demand high-dosage drug exposures and thereby increase the threat of off-target toxicity potential 3. To reverse this unfavorable situation, a great number of efforts have been made to develop new strategies for modulating the concentration of targeted protein 4. Towards this end, translationally modulating protein concentrations has emerged, a process by which intracellular disease-associated proteins can be downregulated via antisense oligonucleotides, RNAi, and the emerging CRISPR-Cas system 3. However, the efficacy of these nucleic acid-based tools relies heavily on the short half-life of the target protein 5. Regarding longer-living ones, a promising technology termed PROTAC (proteolysis-targeting chimera) was developed to fill this gap for targeting pathological protein for post-translational degradation 6.

PROTAC is a class of hetero-bifunctional molecules to kill the distance between the intracellular target protein and E3 ubiquitin ligases, turning out a process of degradation of the target by ubiquitination 6. As a tool for basic research, PROTACs can realize the more direct, tunable, and reversible knockdown of target protein than nucleic acid-based tools can 7. Moreover, the binding domains of PROTACs are confined to cognate active sites of targeted protein 8, thereby permitting the elimination of both established and “undruggable” pathogenesis-related proteins. Therefore, on both the research bench and the clinic bed, PROTACs have received increasing attention 3. Nevertheless, the amount of E3 ligases and targeted proteins utilized to accomplish the target protein degradation have been limited by the lack of identified small-molecule ligands, which severely hindered the applicability and the universality of the PROTAC 3, 4.

Intriguingly, peptide-based PROTAC offers a solution to expand the range of available E3 ligases and targetable proteins by utilizing protein or peptide fragments from identified PPI partners 9. As early as 2001, the first peptidic degron was reported to bridge the methionine aminopeptidase-2 (MetAP-2) with a E3 ubiquitin ligase β-TRCP 10. As a proof of concept, this heterobifunctional peptide verified that targeted protein degradation is a feasible method to suppress the bioactivity of the targeted proteins potently, thereby launching a new avenue for drug research and development 3, 6. Peptide‐based PROTACs, however, showed inadequate *in vitro* activity and hardly any *in vivo* utility, mainly due to their intrinsic lack of cell permeability, proteolytic resistance, and the accumulation in the site of interest 3, 6. While some progress has been achieved in developing cell-permeable and proteolytic-resistant peptide-based PROTACs via chemically-modified peptide sidechain and/or cell-penetrating peptide conjugation 11-14, none have entered into clinical trials or approval for clinical application 3, 15. Therefore, significant challenges remain with regard to proposing a systemic solution to overcome the pharmacological hurdles to peptide-based PROTACs.

Once done, it can be expected that peptide-derived protein degradation not only moves beyond the limitation of the conventional drug R&D that focus on binding site occupancy 3, but also expands a broader range to proteins involved in protein-protein interactions (PPI), which is difficult to be targeted by small-molecule compounds because of the enormous interaction interface 16, 17. Predictably, MDMX (also known as MDM4), a conventional undruggable PPI-involved protein, will be targeted by this emerging strategy. MDMX is a p53- and p73- binding protein that functions in the blockage of their anti-cancer activity. Attenuated p53 and p73 fail to modulate cycle arrest and cell apoptosis upon DNA damage, directly resulting in tumor progression, poor prognosis, and therapy resistance 18. The suppression of p53 and p73 activity by the upregulated MDMX in cancer cells has been the target of drug development for retinoblastoma, pancreatic cancer, colorectal cancer, and breast cancer therapy, yet no MDMX antagonist has been approved for clinical application 18-20. Thus, peptide-derived protein degradation will provide a new idea for MDMX inhibition.

**Figure 1:**
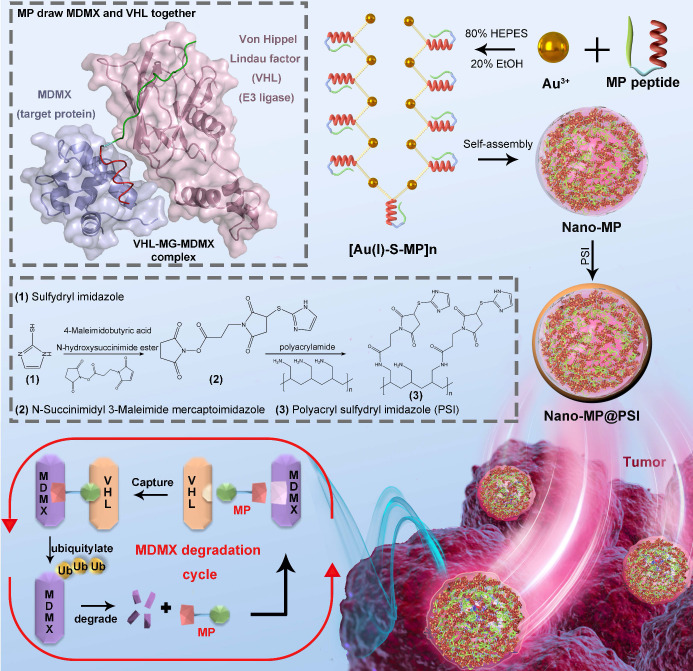
Diagram of the biofunction of the peptide-derived MDMX degrader, MG, and its nanoengineering strategy. A peptide-derived PROTAC was design that is capable of binding in crevices at the surfaces of MDMX and Von Hippel Lindau factor termed VHL (a component of the Cul2-Rbx1-EloB/C-VHL E3 ligase). A nanoengineered gold(I)-peptide cluster (Nano-MP) was synthesized by an aurophilicity-driven self-assembly among Au-peptide precursors [Au(I)-S-MP]_n_. Of note, this gold-derived nanocluster possesses high loading efficiency as the cargo MP themselves were utilized as building blocks.

Here we first of all designed a peptide-derived PROTAC that is capable of binding in crevices at the surfaces of MDMX and Von Hippel Lindau factor termed VHL (a component of the Cul2-Rbx1-EloB/C-VHL E3 ligase) (Figure 1) 21. Because it acts like an adhesive and brings the MDMX and VHL together, this peptide-derived PROTAC was called MDMX predator (MP). Furthermore, to overcome the pharmacological hurdles to peptide-based PROTACs, a nanoengineered gold(I)-peptide cluster (Nano-MP) was synthesized by an aurophilicity-driven self-assembly among Au-peptide precursors [Au(I)-S-MP]_n_. Of note, this gold-derived nanocluster possesses high loading efficiency as the cargo MP themselves were utilized as building blocks (Figure 1). Additionally, Au have shown irreplaceable advantages in essential chemical inertness and subsequent safety 22-24. Moreover, the hypoxic environment of the tumor promotes the glycolysis that produces large amounts of lactic acid and pyruvate, so the extracellular pH in tumor microenvironment (TME) decreases to 6.5 in sharp contrast to pH 7.4 in normal tissues 25, 26. Thus, a macromolecule termed PSI with pH-triggered charge reversal was used to coat the Nano-MP for an enhanced tumor accumulation. As expected, Nano-MP@PSI is highly active against retinoblastoma *in vitro* and *in vivo* through MDMX degradation and subsequent p53 as well as p73 restoration, and it suppressed the tumor progression in a patient-derived tumor xenograft (PDX) model of pancreatic carcinoma harboring the mutant Kras G12D, while showing a highly favorable clearable profile of rapid excretion from the living body. Therefore, this study not only validates MDMX degradation as a promising clinical strategy for anti-cancer therapy, but, more importantly, provides a feasible method to translate peptide-derived PROTACs into a potential drug candidate, and likely reinvigorates their discovery efforts for a great variety of diseases.

## Results and Discussion

### Design, synthesis, and characterization of Nano-MP and Nano-MP@PSI

**Figure 2:**
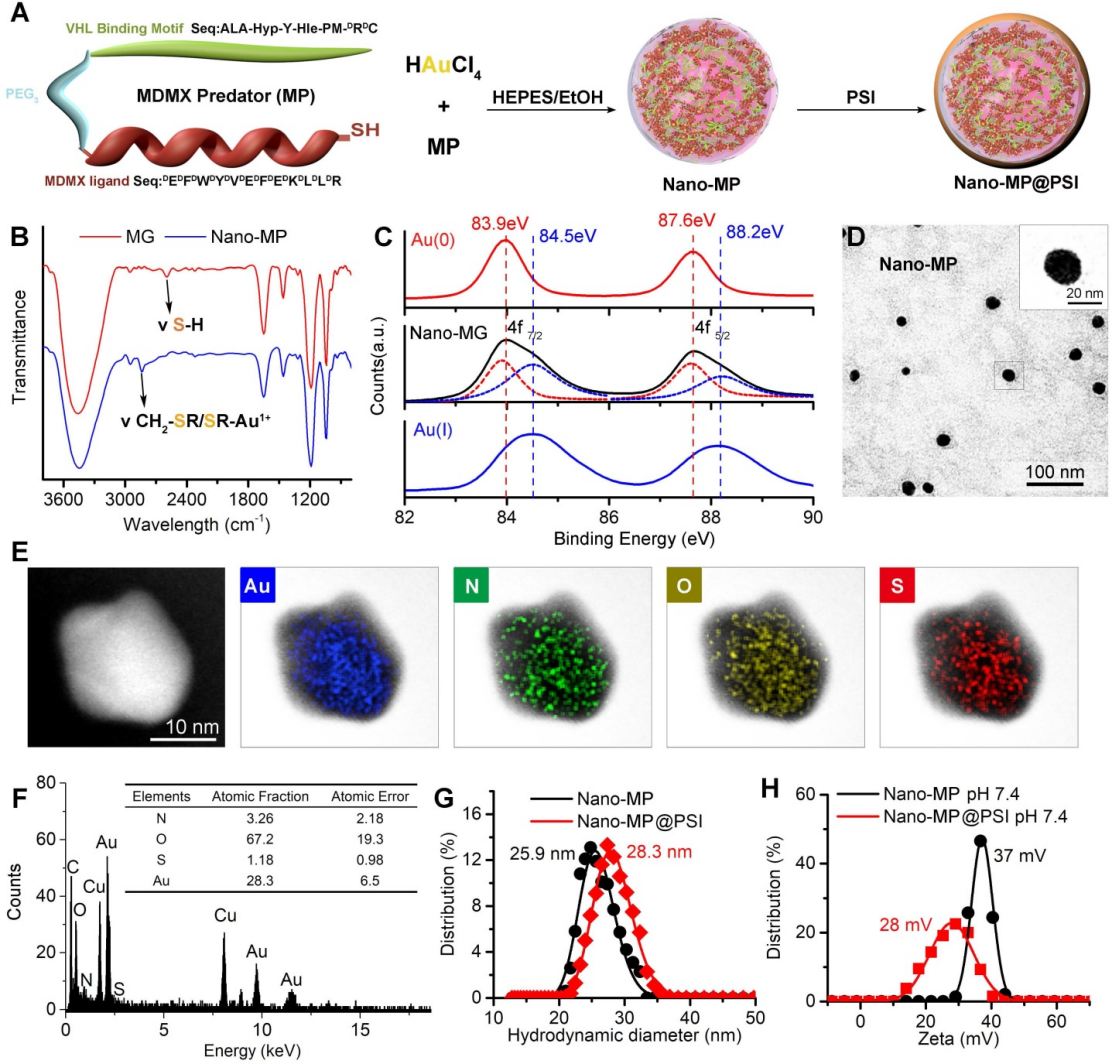
Design, Synthesis and Characterization of Nano-MP and Nano-MP@PSI. **A**, schematic diagram of the constructive process of Nano-MP@PSI. **B**, FTIR spectra. The band at 2650 cm^-1^ has a red shift to 2800 cm^-1^ which was attributed to the stretching vibration of -SH.** C**, Au 4f XPS spectra of Nano-MP (black line), Au(I)-PMI complexes (blue line), and Au(0) nanocrystals (red line).** D**, TEM images of Nano-MP.** E**, elemental analysis image of Au, N, O, S overlay with one representative particle of Nano-MP taken by HRTEM.** F**, EDS quantitative element analysis of Nano-MP. **G** and **H**, hydrodynamic diameter distributions (G) and Zeta potential (H) of Nano-MP and Nano-MP@PSI measured in PBS at pH 7.4.

MP was constructed from three parts: 1) the MDMX binding motif, 2) a flexible tripolymer glycol (PEG_3_) linker, and 3) the hydroxyproline- and homoleucine-containing octapeptide to be recognized by the VHL that is a target recruitment subunit in the Cul2-Rbx1-EloB/C-VHL E3 ubiquitin ligase complex (Figures 1 and 2A). Extra dextrorotatory Cys and Arg residues were introduced to the C terminus of MP for nanocluster construction. Of note, MP was easy to synthesize by solid-phase peptide synthesis (SPSS) following a FMOC chemistry with a yield of ~75% and a purity of >95% (Figure S1), further promoting its application potential. The nanoengineering of the MP was performed by a “two-step and one-pot” reaction in mild conditions. In step 1, 1 mg MP and 1 mg NH_2_-PEG_n_-SH (MW 2000 Da) were mixed into 5 ml 20 mM HAuCl_4_ solution following a 5-min magnetic stirring. Next, in step 2, 5 ml 100 mM HEPES (pH 7.4) were added, and the solution color turned violet. UV-Vis spectroscopy confirmed the formation of the peptide-Au nanoparticle as evidenced by the characteristic of UV absorbance given by the plasma resonance of the gold nanoparticle (540 nm) (Figure S2). The Fourier-transform infrared spectra (FTIR) in Figure 2B showed a shift from the characteristic absorption peak of free thiol in MP spectroscopy to a peak of Gold(I)-Thiolate complex in Nano-MP spectroscopy, indicating that Au ions were bridged by the thiolate sulfur atom in the MG. This intermediate was further characterized by X-ray photoelectron spectroscopy (XPS), in which the Au(4f) peak positions of the Nano-MP were in agreement with results reported for Au(I) ions conjugated to alkanethiols in Cys residue (Figure 2C) 23, 27, 28. These results demonstrated that MP peptide was successfully assembled into a supramolecular gold(I)-thiol-peptide nanocomposite (Nano-MP). Moreover, the transmission electron microscopy (TEM) image and its diffraction pattern revealed the microspheres' spherical and single good dispersion of the Nano-MP (Figure 2D). The overlay chart of elements and TEM image showed the homogeneous distribution of Nitrogen (N), Oxygen (O), Sulphur (S) and Gold (Au) in the nanoparticle (Figure 2E), suggesting the good uniformity of Nano-MP. The energy dispersive X-ray spectroscopy (EDS) analysis exposed that the equally distributed component elements of Nano-MP were coinciding with the constitute of chloroauric acid and peptide (Figure 2F). Collectively, the above results revealed that Nano-MP was successfully constructed as spherical supramolecular gold(I)-thiol-peptide complexes.

To prepare the tumor microenvironment (TME)- responsive nanocluster, the polyacryl sulfydryl imidazole (PSI) was synthesized to pack the Nano-MP. For PSI synthesis, thiol imidazole was first reacted with N-Succinimidyl 6-maleimidohexanoate to produce N-Succinimidyl 3-Maleimide mercaptoimidazole (Figure 1). Next, this activated imidazole was coupled with polyacrylamide (PAA, MW 20000 Da) through the reaction of the amino groups of PAA with the carboxyfluorescein diacetate succinimidyl ester (Figure 1). The product PSI can coat externally on Nano-MP as evidenced by the increased hydrodynamic diameter (Figure 2G) and decreased ZETA potential at pH 7.4 (Figure 2H). These results provided enough evidence that the PSI coating of supramolecular complexes termed Nano-MP@PSI was successfully constructed. Of note, PSI coating didn't change the spherical morphology of Nano-MP (Figure S3).

### The PSI modification further increased the blood-circulation time and tumor-specific accumulation

**Figure 3:**
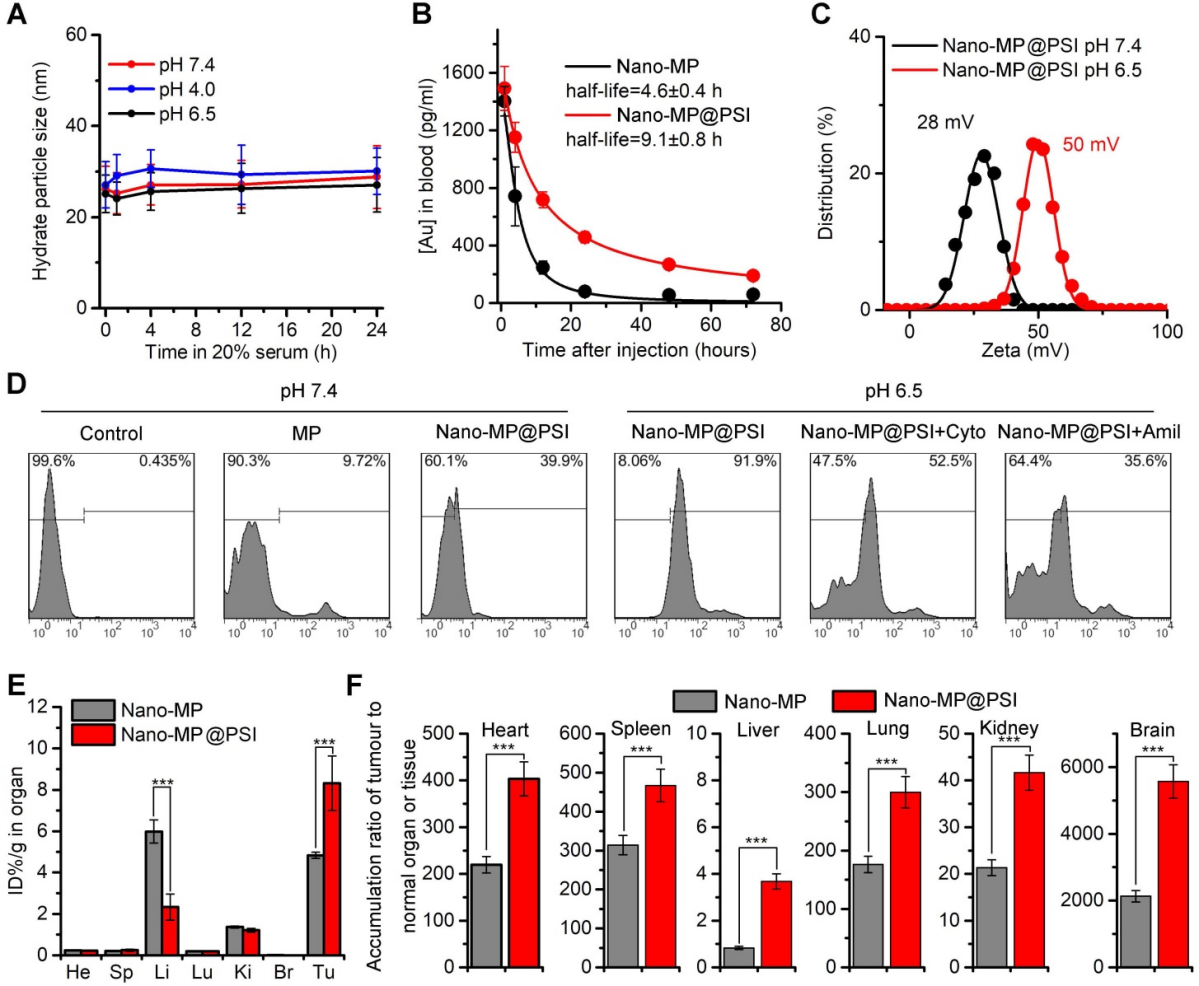
The PSI modification further increased the blood-circulation time and tumor-specific accumulation.** A**, colloidal stability of Nano-MP@PSI suspending in PBS containing 20% FBS at pH 4.0, 6.5 and 7.4 measured by DLS. **B**, blood-circulation curves of Nano-MP and Nano-MP@PSI in healthy C57/B6 mice by measuring the concentrations of Au in blood at different time points post injection. The error bars were based on the standard deviations (SD) of triplicate samples. **C**, Zeta potential of Nano-MP@PSI measured in PBS at pH 7.4 and 6.5.** D**, cell uptake analysis of WERI-Rb-1 cells in control condition (PBS control) and after pretreatment with Cytochalasin D (Cyto, 2 µM) or Amiloride (Amil, 3 mM) for 6 h before Nano-MP@PSI incubation. **E**, organ distribution of Nano-MP and Nano-MP@PSI in BALB/c mice after systemic injection. Serial sacrifices were carried out at 6 h after dosing. Several organs/tissues, including heart, liver, spleen, lung, kidney, brain and tumor were isolated to determine gold concentrations by ICP-MS. He, heart; Li, liver; Sp, spleen; Lu, lung; Ki, kidneys; Br, Brain. **F**, tumor-to- organ ratios for Nano-MP@PSI and Nano-MP at 6 h after dosing. The data were mean ± SD.

The blood-circulation time and tumor-specific accumulation are vitally important for the potency of anti-tumor nanomedicine. As expected, Nano-MP@PSI possessed good colloidal stability. As shown in Figure 3A, Nano-MP@PSI remained monodispersed and showed hardly any change in size over the entire 24 hours in standard 1X PBS solution, blending 20% fetal calf serumat pH 4.0, 6.5, and 7.4. This suggested that our PSI coating supramolecular gold(I)-thiol-peptide complexes may shelter from risk in the infaust aggregation and consequent reticuloendothelial system uptakes, thereby resulting in an optimized blood circulation time. To validate it, we found the detection and quantitation of ^197^Au by the inductively coupled plasma mass spectrometry (ICP-MS) in the blood extracted from healthy C57L/B6 mice. Time-dependent ICP-MS measurements in the blood yielded metabolic kinetics in support of the prolonged blood-circulation time of Nano-MP@PSI in comparison to Nano-MP (Figure 3B). Additionally, >90% peptide maintained in the particle after a 72h incubation in a PBS buffer containing 20% blood serum, suggesting the integrality of Nano-MP@PSI during the circulation (Figure S4).

Moreover, the dissociation constant (pKa) of imidazole ranges from 6 to 7, so the PSI would be further protonated at pHe in TME. As expected, the ZETA potential of Nano-MP@PSI shifted form 28 mV at pH 7.4 to 50 mV at pH 6.0 (Figure 3C). Nanoparticles with a strong positive charge can electrostatically attract to the cancer cell membrane with a negative charge, which subsequently triggers cellular internalization 22, 28, 29. Thus, the protonated Nano-MP@PSI in TME should possess the enhanced cytomembrane penetrability in contrast to unprotonated ones in a normal physiological environment at pH 7.4. To ascertain the ability of Nano-MP@PSI to traverse the membrane of cancer cells, fluorescein isothiocyanate (FITC) was N-terminally conjugated to the MG, and the cellular uptakes of ^FITC^MG and Nano-^FITC^MG@PSI were examined by flow cytometry analysis after 6-hour incubations. As a result, Nano-MP@PSI showed enhanced internalization into cancer cells at pH 6.5 in comparison with the same at physiological pH (Figure 3D). Additionally, the preincubation of 3mM Amiloride (micropinocytosis inhibitor) and 2 μM cytochalasin D (actin inhibitor) suppressed the Nano-^FITC^MG@PSI internalization at pH 6.5, suggesting an actin-dependent micropinocytosis. This pH-responsive cellular internalization will arm Nano-MP@PSI with the enhanced tumor accumulation. To validate it, ICP-MS was used to detect and quantify ^197^Au in the organs and tumors. As shown in Figure 3E, biodistributions of two nanoparticles were presented as the percentage of Injected Dose in per gram of organ or tumor (ID%/g) in Figure 3E, and Nano-MP@PSI, as expected, exhibited an increased tumor accumulation 6 hours after injection. Through further analysis of this ^197^Au distribution, we found that all ratios of tumor to normal organ for Nano-MP@PSI were superior to that for Nano-MP (Figure 3F), indicating that the PSI modification further increased the tumor-specific accumulation of Nano-MP.

### Nano-MP@PSI can GSH-trigger release cargo and be excreted from the body

**Figure 4:**
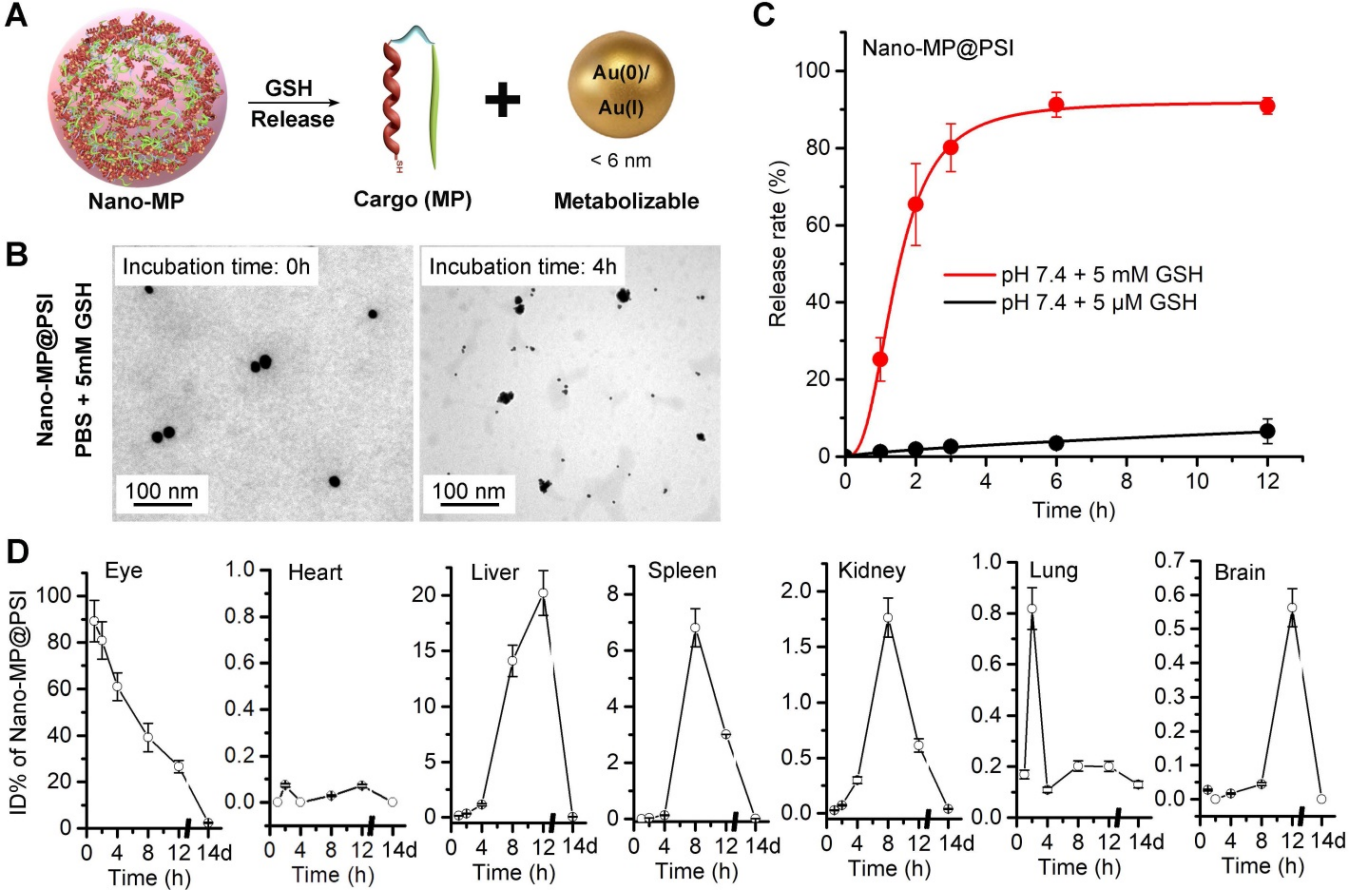
Nano-MP@PSI can GSH-triggered release cargo and be excreted from the body. **A**, diagrammatic drawing of the GSH-triggered release of cargo (MG) and size change of Nano-MP. **B**, TEM images of Nano-MP@PSI at 0h and 4h after 5 mM GSH incubation at pH 7.4. **C**, peptide release from Nano-MP@PSI under 5mM GSH or 5μM solutions at pH 7.4. Peptide release was quantified by HPLC, and the date was showed by Mean±SD. **D**, tissue kinetics of Nano-MP in BALB/c mice after intravitreal injection. Serial sacrifices were carried out at 1h, 2h, 4h, 8h, 12h and 14 days after dosing. Several organs/tissues, including eye, heart, liver, spleen, lung, kidney and brain were isolated to determine gold concentrations by ICP-MS. The data were shown as mean ± SD.

As in our previous reports, Au(I)-alkanethiols bond would fracture in response to the high glutathione (GSH) concentration in cancer cells, while remaining stable in the extracellular environment 22, 23, 28. When stimulated, Nano-MP can disassemble into an ultra-small nanoparticle of the size of only ~5 nm in response to intracellular reducing environments (Figure 4A), which was amply demonstrated by the TEM image (Figure 4B) and DLS measurement (Figure S5) after a 5mM GSH treatment. During this disassembly, a ~90% MG release can be found upon 3-hour incubation in a simulated intracellular environment solution containing standard PBS and 5 mM GSH at pH 6.0 (Figure 4C). This is in sharp contrast to a <10% release after a 12-hour incubation in a simulated extracellular environment solution including 5 μM GSH at pH 7.4 (Figure 4C). In a word, this supramolecular gold(I)-thiol-peptide nanocomposite is a stable and viable platform for intracellular delivery and controlled release of peptide cargo.

Clearable nanoparticles have recently emerged as a new class of engineered nanomedicine that can purposefully deliver cargo to the site of interest while off-target nanoparticles and work-performed nanocarriers can be rapidly eliminated out of the body with the urine and/or systematically removed through the mononuclear phagocytic system 30, 31. These nanoparticles have shown huge potentials to weaken the systemic toxicity by averting the non-specific accumulation in healthy tissues and organs 32. With their ultra-small size (< 6 nm), these nanoparticles can be efficiently excreted from the body after the systemic and/or partial administration 33, 34. Of note, such an ultra-small size will even endow our nanomedicine with penetrability across blood-eye barrier 35, 36. To further challenge the cleanability of the Nano-MP@PSI, we mass-spectrographically monitored the disturbed kinematics of Nano-MP in mice after intravitreal injection, and the detection and quantitation of ^197^Au in tissues and organs were performed using inductively coupled plasma mass spectrometry (ICP-MS). Time-dependent ^197^Au measurements in the eye, expressed as ID%, yielded metabolic kinetics whereby over 70% of ^197^Au were eliminated from the eye after 8 hours and nearly all of them were emptied after 2 weeks (Figure 4D). Furthermore, hardly any ^197^Au were found in the heart, liver, spleen, kidney, lung, or brain after 2 weeks of injection (Figure 4D). Notably, the liver and spleen undertook the most metabolic tasks of ^197^Au (Figure 4D), implying that Nano-MP@PSI eliminated out of the body in an MPS (mononuclear phagocytic system)-dependent fashion. Collectively, these results suggested that Nano-MP is a clearable nanoparticle.

### Nano-MP@PSI Induces MDMX degradation and subsequent p53 as well as p73 restoration

**Figure 5:**
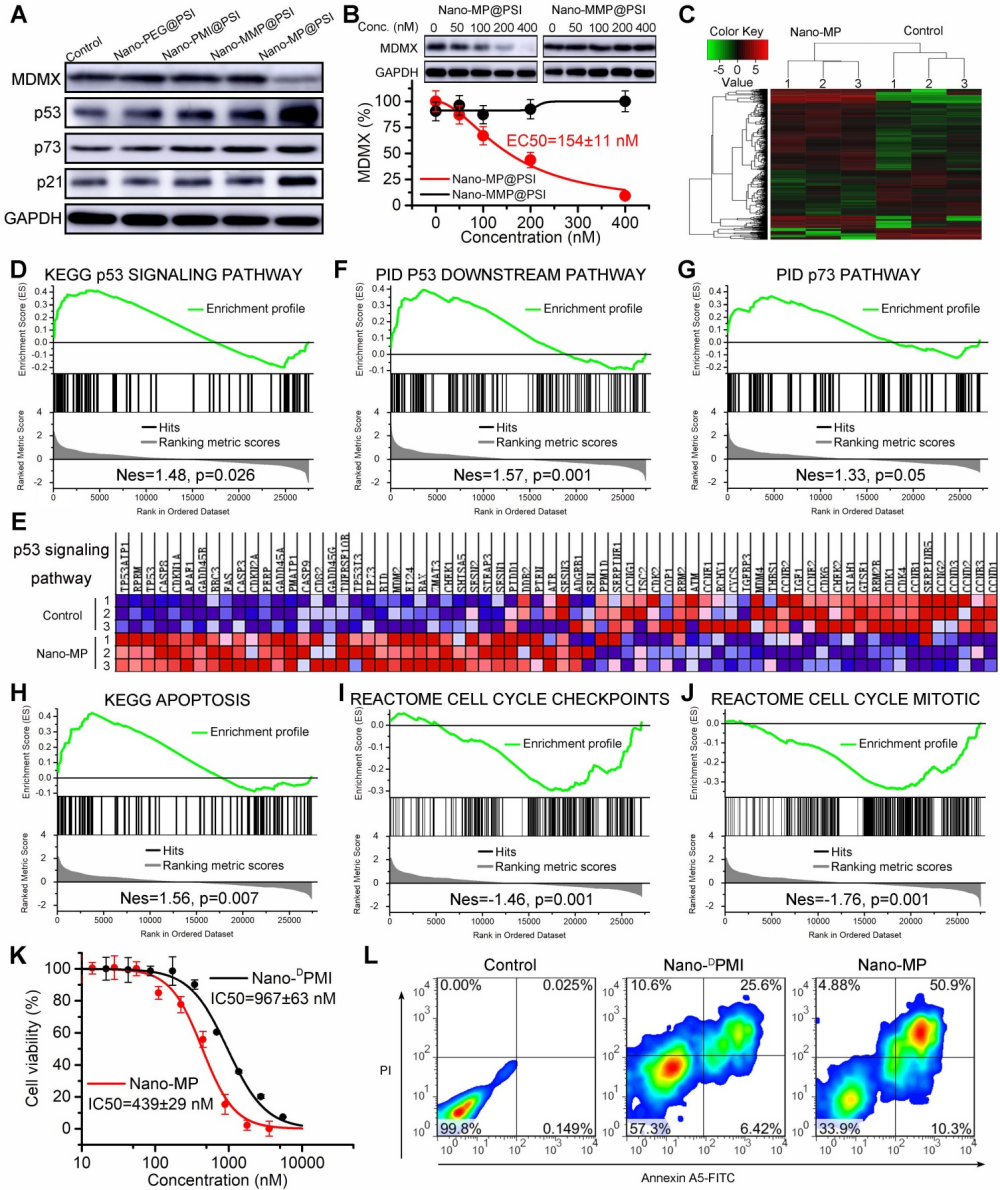
Nano-MP@PSI induces MDMX degradation and subsequent p53 as well as p73 restoration. **A**, WERI-Rb-1 cells were treated with Nano-MP@PSI, Nano-^D^PMI@PSI, Nano-PEG@PSI and Nano-MMP@PSI for 6 h at the dosage of 100 nM, and western blot was performed to analyze the expressions of MDMX, p53, p73 and p21 proteins. GAPDH was used as loading control. **B**, WERI-Rb-1 cells were treated with the indicated concentration of the Nano-MP@PSI for 6 h to detect the dose dependent degradation of MDMX. **C**, hierarchical clustering of genes differentially expressed in WERI-Rb-1 cells after exposure to 1 µM Nano-MP@PSI for 12 hours compared with mock-treated cells (n = 3). **D&E**, Gene set enrichment analysis (GSEA, **D**) and hierarchical clustering of genes (**E**) in p53 signaling pathway. **F-J,** GSEA showing the p53 downstream pathway, p73 pathway, apoptosis, cell cycle checkpoints or cell cycle mitotic signatures differentially expressed in response to Nano-MP@PSI. KEGG, Kyoto Encyclopedia of Genes and Genomes; PID, Pathway Interaction Database; Nes, normalized enrichment score. **K**, Dose-dependent curves of indicated samples against WERI-Rb-1 cells after 48 h incubation measured by CCK8 assay (n =4, mean ± s.d.). WERI-Rb-1 carried wildtype p53 and over-expressed MDMX. **L**, Apoptosis levels of Nano-MP@PSI and Nano-^D^PMI@PSI on WERI-Rb-1 cells were determined by Annexin V-PI staining and flow cytometric analysis.

Once synthesized, the ability of the Nano-MP@PSI to induce MDMX degradation in cells was first evaluated by immunoblotting (Figure 5A). By comparison, a blank carrier control termed Nano-PEG@PSI was synthesized as a negative control, in which MP peptide was changed to mercapto polyethylene glycol (PEG, MW:2000). Additionally, two control MDMX degraders were used as positive controls: The one termed Nano-^D^PMI@PSI synthesized by a modified MP peptide truncated VHL ligand. The other, termed Nano-MMP@PSI, synthesized by a modified MP peptide containing a VHL ligand with an inverted chiral center. The retinoblastoma cell line WERI-Rb-1 was selected for the initial evaluation of Nano-MP given the over-expressed MDMX and wild-type status of p53 as well as p73 of this cell line 37, 38. As expected, 100 nM Nano-MP@PSI *in vitro* down-regulated MDMX after a 6-hour incubation, while no obvious change in MDMX levels could be found in cells after incubations with Nano-MMP@PSI, Nano-PEG@PSI, or Nano-^D^PMI@PSI (Figure 5A). As a result, Nano-MP@PSI stabilized p53, p73, and p21 more actively than Nano-MMP@PSI and Nano-^ D^PMI@PSI did (Figure 5A). Additionally, the results were proved again by the proteomics measured by label-free mass spectrometric analysis, in which Nano-MP@PSI up-regulated proteins in the p53 signaling pathway with more action than Nano-MMP@PSI and Nano-^ D^PMI@PSI did (Figure S6).

Moreover, we found that Nano-MP@PSI induced the MDMX degradation in a dose-dependent manner with a DC50 of 154 nM in WERI-Rb-1 cells after a 6-hour incubation (Figure 5B). Significantly, to eliminate interference from transcription, a ribosome inhibitor termed CHX was used to pretreat the cell for 12 hours before the Nano-MP@PSI incubation. In addition, to explore the degradative pathway of MDMX, a proteasome inhibitor MG132 and an E1 ubiquitinase inhibitor PYR41 were used to suppress the MDMX degradation from 200 nM Nano-MP@PSI. As expected, 12-hour preincubation of MG132 and PYR41 totally inhibits the action of Nano-MP@PSI (Figure S7A). In sharp contrast, neither a chloroquine (a lysosome inhibitor) nor NH_4_Cl (an autophagy inhibitor) had any effect on the function of Nano-MP@PSI (Figure S7B). These results demonstrated that Nano-MP@PSI induced MDMX degradation in an ubiquitin-dependent degradation pathway other than autophagy/lysosome-mediated degradation.

At a transcriptional level, Nano-MP@PSI triggered 776 differentially expressed genes after 24-hour incubation on WERI-Rb-1 cells as evidenced by RNA sequencing and subsequent clustering analysis (Figure 5C). The further gene set enrichment analysis (GSEA) exposed that p53-regulated gene sets were significantly upregulated in the Nano-MP@PSI-treated cells compared to the mock-treated cells (Figures 5D-F). Meanwhile, we conducted GSEA to identify the other gene sets related to p53 and found 4 significantly enriched pathways: p73 signaling (Figure 5G), apoptosis (Figure 5H), cell cycle checkpoints (Figure 5I), and cell cycle mitosis (Figure 5J). Collectively, GSEA results demonstrated that Nano-MP transcriptionally activated the p53 signaling pathway as well as the relevant apoptosis and restrained cell cycle pathways. As a result, Nano-MP@PSI significantly suppressed the proliferation of WERI-Rb-1 cells *in vitro* (Figure 5K) and induced their apoptosis (Figure 5L) as well as cell cycle arrest (Figure S4). More importantly, Nano-MP showed intensive anti-cancer activity in comparation to Nano-^D^PMI (Figures 5K, L and S4). Together, these results demonstrate that Nano-MP@PSI degrades MDMX and displays on-target p53 and p73 pathway activity *in vitro*.

### Nano-MP potently and safely suppressed retinoblastoma in an orthotopic xenograft mouse model

**Figure 6:**
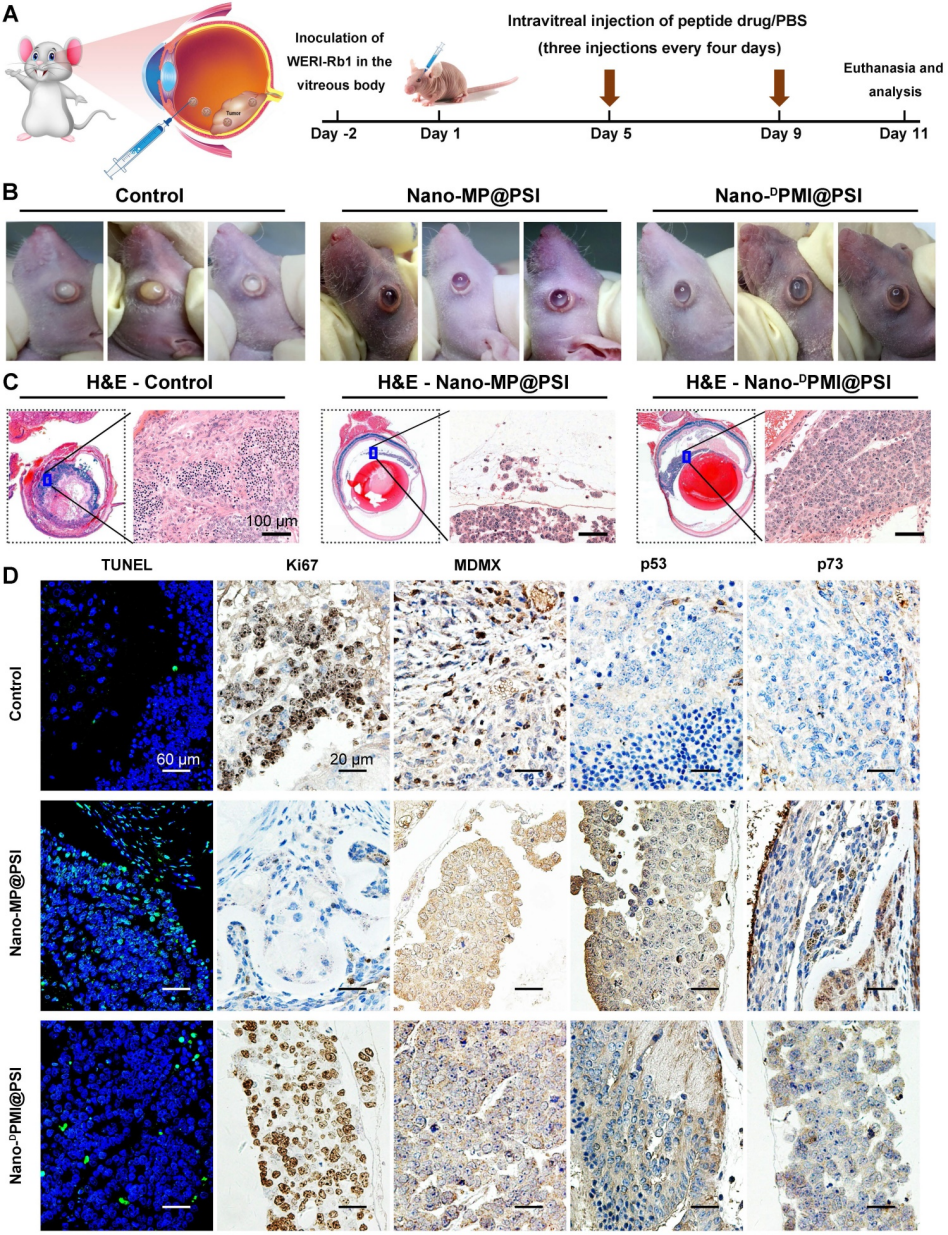
Nano-MP@PSI potently suppressed retinoblastoma in an orthotopic xenograft mouse model. **A**, Schematic diagram of therapeutic treatments. Athymic nude mice (BALB/c) bearing WERI-Rb1xenograft tumors were established and randomly divided into three groups (n = 5/group). Mice were then treated by intraperitoneal injection at day 1, 5, 9 of PBS, Nano-MP@PSI (1.5 mg/kg) or Nano-^D^PMI@PSI (1.5 mg/kg). **B**, Photographic images from BALB/c mice bearing WERI-Rb1 xenograft tumors with the indicated treatments. **C**, Representative tumor sections after the 11-day treatment staining by H&E (X 200). **D**, Representative TUNEL and IHC images for Ki67, MDMX, p53, and p21 in tumor sections (scale bar: 60 µm).

Retinoblastoma currently has the highest incidence of primary intraocular malignancy in children, which not only results in blindness, but also poses a threat to the lives of young patients 39, 40. Thus, it is an appropriate and sensible intraocular disease model to challenge Nano-MP@PSI. 1×10^5^ WERI-Rb-1 cells were seeded in the subretinal space of one eye of the Balb/c nude mice to establish an orthotopic xenograft retinoblastoma mouse model (Figure 6A) 39, 41. Retinoblastoma-bearing mice were randomized into three groups (n=3/group) and received normal saline (NS) (Control), Nano-MP@PSI, or Nano-^D^PMI@PSI treatment by intravitreal injection according to the schedule in Figure 6A. At day 11, it was found that Nano-MP@PSI treatments significantly suppressed the progression of retinoblastoma compared to the controls (Figure 6B). Meanwhile, Nano-MP@PSI showed better therapeutic effect than Nano-^D^PMI@PSI (Figure 6B). These results were further proved by the H&E staining, in which the tumor areas were shrunk by Nano-MP@PSI treatment in sharp contrast to the control and Nano-^D^PMI@PSI treatment (Figure 6C). Moreover, TUNEL immunofluorescence analysis of the apoptosis and ki67 immunohistochemical (IHC) assay to proliferation further supported these results (Figure 6D). Additionally, as expected, we found a significant decrease in MDMX levels in the Nano-MP@PSI-treated tumors (Figure 6D), suggesting its role for MDMX degradation. As a result, the restoration of p53 and p73 further validates the anti-cancer activity and mechanism of Nano-MP@PSI (Figure 6D). Furthermore, no obvious change of mice weight (Figure S5A) and pathological morphology of viscera (Figure S5B) can be found in Nano-MP@PSI- or Nano-^D^PMI@PSI-treated mice compared to NS-treated mice, indicating the absence of any acute toxicity of the nanoparticles themselves. In brief, Nano-MP@PSI effectively and safely suppressed retinoblastoma development by degrading MDMX.

### Nano-MP@PSI induces regression in the PDX model of pancreatic carcinoma

**Figure 7:**
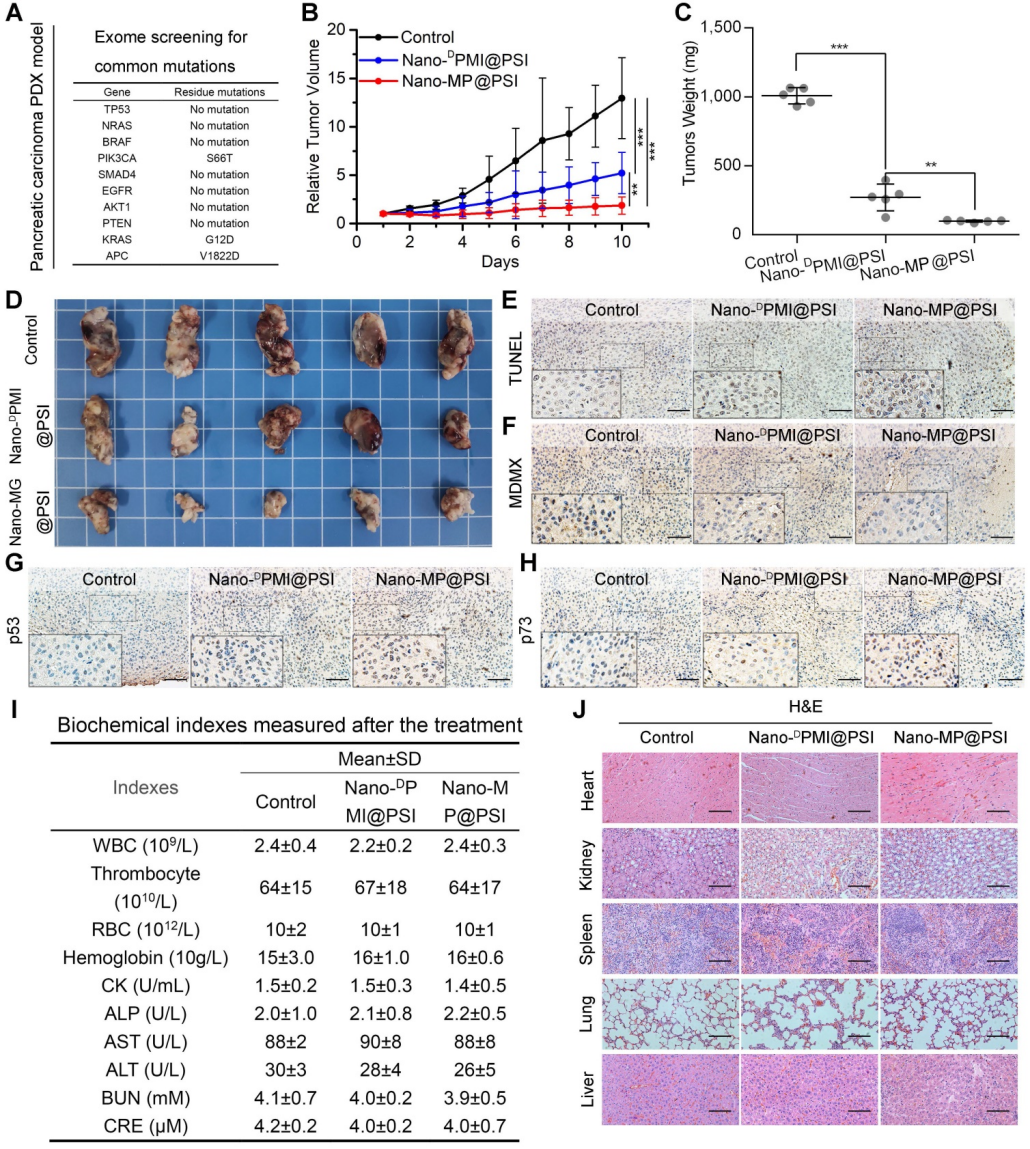
Nano-MP@PSI induces regression in the PDX model of pancreatic carcinoma. **A**, the exome sequencing for pancreatic carcinoma PDX tumor. **B**, tumor growth curves in nude mice subcutaneously inoculated with PDX tumor in fossa iliaca. Data are presented as mean ± s.e. (n =5). **C**&**D**, weights (C) and Photos (D) of the tumors excised at the end of the experiment. *p* values were calculated by t-test (*, p <0.05; **, p <0.01; ***, p <0.001). **E**-**H**, representative images of TUNEL (E) and immunohistochemical (IHC) images of MDMX(F), p53(G) and p73(H) staining for tumor tissue (scale bar: 50 µm). **I**, safety evaluation of Nano-MP and Nano-^D^PMI. The count of RBC, hemoglobin, WBC, thrombocyte and eosinophils, the activities of heart enzymes CK, the activities of two liver enzymes ALT and AST and the activities of two kidney enzymes ALT and AST in mice blood were measured after the 10-day treatment. **J**, representative images of H&E-stained heart, liver, spleen, lung and kidney sections in mice with the indicated treatments (scale bar: 50 µm).

Mouse models with monocellular tumor xenografts have been the standard tool for oncology research for decades 42. A growing number of studies, however, have proven that the principal histologic, genetic, and microenvironmental characteristics of monocellular tumors and actual tumors have always diverged substantially 43. Hence, patient-derived xenograft (PDX) models where xenograft primary or metastatic tumors directly from the patient were introduced into severe combined immunodeficient mice became increasingly popular for therapeutic screening, biomarker discovery, and especially the preclinical evaluation of drugs in the last decade 43. Moreover, it has been proven that subcutaneously inoculated PDX models of pancreatic carcinoma retain the histological and stromal features of the parental tumor 44, 45. Thus, to further investigate the therapeutic efficacy of Nano-MP@PSI, we comparatively examined its effect with Nano-^D^PMI@PSI on tumor growth, tumor weight, tumor cell apoptosis, and levels of MDMX, p53 as well as p21, using NOD/SCID mice bearing the first passage of a high-grade malignant PDX tumor of pancreatic cancer derived from a surgically resected residual tumor that harbored wild-type p53 and mutant KRAS, APC, and PI3KCA (Figure 7A). Mice bearing the PDX tumor with a volume of 100 ± 25 mm^3^ were randomly assigned to respectively receive intravenous injection of NS (control), Nano-MP@PSI, or Nano-^D^PMI@PSI. During the 10-day treatments, mice in the control group experienced a greater than 13-fold increase in tumor volume, while 1.5 mg/kg peptide equivalent dose of Nano-MP@PSI or Nano-^D^PMI@PSI inhibited this tumor growth by 85.7% and 59.7%, respectively (Figure 7B). The mass (Figure 7C) and image (Figure 7D) of the isolated PDX tumor appears to support the powerful anti-cancer activity of Nano-MP@PSI. Furthermore, terminal deoxynucleotidyl transferase-mediated nick end labeling (TUNEL) analysis of residual tumors in different treatment groups following the Nano-MP@PSI treatment showed significantly increased cell apoptosis compared with PBS or Nano-^D^PMI@PSI treatment (Figure 7E). Immunohistochemical staining of tumor tissue sections revealed significant downregulation of MDMX (Figure 7F) and obvious upregulation of p53 (Figure 7G) as well as p73 (Figure 7H) in the tumor tissues treated with Nano-MP@PSI. Moreover, neither Nano-MP@PSI nor Nano-^D^PMI@PSI treatment resulted in significative change in body weights (Figure S6), blood biochemical indexes (Figure 7I), and pathological section of viscera (Figure 7J) in comparison to the mock treatment, indicative of their safety. Together these results indicate that Nano-MP@PSI possessed enhanced therapeutic efficacy in KRAS mutant pancreatic cancer through degrading MDMX and subsequent p53 as well as p73 restoration, while keeping a highly favorable biosafety profile.

Compared with small-molecule-derived PROTAC technology, peptide-derived degraders have an irreplaceable advantage to extend the range of ligands for E3 ligases and targeted proteins utilized to accomplish the target protein degradation. However, peptide therapeutics always suffered from their intrinsic lack of cell permeability, proteolytic resistance, and the accumulation in the site of interest. In this work, we made unprecedented use of the PROTAC strategy to design a peptide degrader targeting MDMX and nanoengineered it into a supramolecular gold(I)-thiol-peptide complex termed Nano-MP with potential as a drug candidate for malignant tumors with over-expressed MDMX. We conclude that Nano-MP specifically induces the degradation of MDMX in an ubiquitination-dependent manner and subsequent restores the antineoplastic function of p53 and p73. As expected, Nano-MP is highly active against retinoblastoma *in vitro* and *in vivo*, while showing a highly favorable clearable profile of rapid excretion from the living body after intraocular injection. In addition, the imidazole-modified version of Nano-MP exhibited the tumor-specific accumulation upon systemic injection and effectively suppressed the tumor progression in a PDX model of pernicious pancreatic carcinoma that harbors the mutant Kras G12D. In a manner of speaking, Nano-MP has tremendous potential as a candidate drug to target MDMX. Of note, some small molecules have been successfully used as PROTACs to target MDM2 — a homologous protein of MDMX with the same function to inhibit the anti-cancer activities of p53, but that probably suffered from the limited action ascribed to the compensatory mechanism of MDMX upregulation 7, 46, 47. Moreover, to our knowledge, peptide-based PROTACs targeting the MDMX or MDM2 only showed limited cellular and creaturely function. Thus, this study not only filled the gap of the MDMX degrader, but also extended PROTAC technology to peptides.

## Conclusions

Nano-MP represents a successful combination of peptide chemistry and nanotechnology to design a PROTAC molecule and shows the enormous potential of peptide-derived nano-degraders in targeting the pathogenesis-related proteins involved in PPIs. The strategy reported here will be easily extended to other peptide-protein interactions, significantly bridging the chasm between peptides and PROTACs. Taken together, this study not only validates MDMX degradation as a promising clinical strategy for anti-cancer therapy, but, more importantly, provides a feasible method to translate peptide-derived PROTACs into a potential drug candidate, and likely reinvigorates discovery efforts aimed at “undruggable” targets for a great variety of diseases.

## Supplementary Material

Supplementary figures and tables.
