# Supplementary material for: A nano-predator of pathological MDMX construct by clearable supramolecular gold(I)-thiol-peptide complexes achieves safe and potent anti-tumor activity
Source: Theranostics. 2021 May 3;11(14):6833–46. doi: 10.7150/thno.59020 (PMC8171083; doi:10.7150/thno.59020)
Supplement: Supplementary file 1 — Supplementary figures and tables. [file thnov11p6833s1.pdf]

# Supplementary materials

# A nano-predator of pathological MDMX construct by clearable supramolecular gold(I)-thiol-peptide complexes achieves safe and potent anti-tumor activity

Siqi Yan [1,2,†], Jin Yan[3,*,†], Dan Liu[1,†], Xiang Li[4,†], Qianyan Kang[2], Weiming You[3], Jinghua Zhang[5], Lei Wang[6], Zhiqi Tian[7], Wuyuan Lu[8,*], Wenjia Liu[6,*] and Wangxiao He[1,6,*]

1. Department of Talent Highland, The First Affiliated Hospital of Xi'an Jiaotong University, Xi'an 710061, China.

2. Ophthalmology Department, The First Affiliated Hospital of Xi'an Jiaotong University, Xi'an 710061, China.

3. National & Local Joint Engineering Research Center of Biodiagnosis and Biotherapy, The Second Affiliated Hospital of Xi'an Jiaotong University, Xi'an 710004, China.

4. School of Pharmacy, Second Military Medical University, Shanghai 200433, China.

5. School of Public Health, Xi'an Jiaotong University Health Science Center, Xi'an 710061, China.

6. Institute for Stem Cell & Regenerative Medicine, The Second Affiliated Hospital of Xi'an Jiaotong University, Xi'an 710004, China.

7. Department of Cancer Biology, University of Cincinnati College of Medicine, Cincinnati, 45267 OH, USA.

8. School of Basic Medicine, Fudan University, Shanghai 20433, China.

[†] These authors contributed equally.

[*] Corresponding authors:

Email: yanjin19920602@xjtu.edu.cn (J. Yan)

Email: wlu@ihv.umaryland.edu (W. Lu)

Email: wenjia@xiterm.com (W. Liu)

Email: hewangxiao5366@xjtu.edu.cn (W. He)

# 1. Experimental Section

**General remarks**

All synthetic peptide sources were obtained from ChinaPeptides Co.,Ltd. All other chemicals used in this study were purchased from Sigma-Aldrich unless otherwise specified. $HAuCl_4 \cdot XH_2O$ was purchased from Aladdin Chemicals. All products were used as received without further purification.

**Synthesis of MG peptide (VHL Binding Motif and MDMX ligand)**

All peptides were synthesized on appropriate resins on an CS bio 336X automated peptide synthesizer using the optimized HBTU activation/DIEA in situ neutralization protocol developed by an HBTU/HOBT protocol for Fmoc-chemistry SPPS. After cleavage and deprotection in a reagent cocktail containing 88% TFA, 5% phenol, 5% H2O and 2%TIPS, crude products were precipitated with cold ether and purified to homogeneity by preparative C18 reversed phase HPLC. The molecular masses were ascertained by electrospray ionization mass spectrometry (ESI-MS).

**Synthesis of Nano-MG**

First, 2 mg of MG peptide was completely dissolved in a solution containing 500μL ethanol and 1.25mL $ddH_20$. After that, an aqueous solution of tetrachloroauric acid (HAuCl4·XH2O, 1 mL, 10 mM) was mixed with 500μL $NH_2$-PEG-SH (MW: 2000, 4mg/ml in deionized water) and 2.25

ml HEPES (100mM, pH 7.0). Then it mixed with the prepared solution containing 2.25 ml deionized water and 2.25ml HEPES (100mM pH 7.0), sonicate for 10 min. Finally, removed the excess reactants by dialysis tubing (cutoff, 10 KDa) and washed twice by distilled water.

**Fabrication of Nano-MG@PSI**

For PSI synthesis, thiol imidazole was first reacted with N-Succinimidyl 6-maleimidohexanoate to produce N-Succinimidyl 3-Maleimide mercaptoimidazole (Molar Ratio: 1:1). Next, this activated imidazole was coupled to the polyacrylamide (PAA, MW 20000 Da) through the reaction of the amino groups of PAA with the carboxyfluorescein diacetate succinimidyl ester. Then the prepared PSI and Nano-MG (volume ratio of PSI:Nano-MG=1:200) were mixed and ultrasonicated at 30 W for 2 min. The mixture was stored at 4°C for further analyses.

**Physicochemical properties of Nano-MG and Nano-MG@PSI**

The morphology and lattice structure were observed on high-resolution transmission electron microscopy (HRTEM), which was performed on a Talos F200X. One portion of the pellet was placed onto a carbon-coated copper grid for imaging with high-resolution transmission electron microscopy (HRTEM) and selected area electron diffraction (SAED). Energy-dispersive spectroscopy (EDS) analysis was performed on the nanoparticles formed from $Au^{3+}$ at 20 kV accelerating voltage and 133 eV resolution on a scanning area of 1 × 1 μm using an EX-250 spectrometer. The hydrodynamic size distribution (1 mg/mL in PBS, 1 mL) was obtained from the dynamic light scattering (DLS) measurement (Malvern Zetasizer Nano ZS system). For Zeta potential measurement, the nanoparticles (1 mg/mL, 1 mL) were incubated with PBS at different

pH at 37 ºC for 30 min, and measured by dynamic light scattering (DLS). The surface chemical structure of modified nanocrystals was evaluated by Fourier transform infrared (FT-IR) spectroscopy (Nicolet 6700) and UV−vis absorption spectra (Shimadzu 3000 spectrophotometer).

**GSH-responded drug release**

To test GSH-responded drug release, Nano-MG were dissolved in PBS buffer (pH 7.4) containing 5 mM or4 5 μm glutathione (GSH), and the nanoparticles were then removed by 14000 g centrifuge. Following this, the supernatants were quantified by HPLC and authenticated by ESI-MASS. Separations were performed at a flow rate of 1 mL/min with a gradient from 5 to 65% of B in 30 min (eluent A: 0.1% TFA/H2O, eluent B: 0.1% TFA in $CH_3CN$).

**Cell culture and viability analysis**

WERI-Rb-1 cell carrying wild-type p53 was purchased by ATCC, and maintained in RPMI1640 medium with 10% FBS. All cells were maintained at 37°C in an atmosphere of 5% $CO_2$. The *in vitro* cytotoxicity was measured using a standard Alamar blue (Thermo Fisher scientific) assay in the above cell lines. Cells were plated in 96-well plates at a density of 2500 cells/well (100 uL). After 48 h, cells were treated with prescribed samples at the indicated concentrations and times, respectively. All cells were maintained at 37°C in an atmosphere of 5% $CO_2$.

**Apoptosis and cell cycle analysis**

Necrosis/apoptosis was evaluated by flow cytometry using the FITC Annexin V Apoptosis

Detection Kit (BD Biosciences). Briefly, cells were treated with 1μM Nano-MG and 1μM Nano-$^{D}$PMI for 48 h. Cells were then harvested, washed twice with cold PBS, and resuspended in 1×binding buffer at a concentration of $1\times10^{6}$ cells/ml. One hundred microliters of the solution ($1\times10^{5}$ cells) was transferred to a 5-ml culture tube, followed by addition of 5 μl of FITC Annexin V and 5 μl of PI. After gentle vortexing and a 15-min incubation in the dark at room temperature, 400 μl of 1× binding buffer was added to the tube, and cells were analyzed by FACS.

Cells were first serum starved for 12 h, and then treated with Nano-MG and Nano-$^{D}$PMI for 6 h. Next, cells were harvested, washed twice in PBS, and fixed in 70% ethanol on ice for at least 30 min. After that, cells were stained with propidium iodide (PI) solution (50 μg/ml PI, 50 μg/ml RNase A, 0.1% Triton-X, 0.1mM EDTA). Cell cycle distributions were then analyzed based on DNA contents by a flow cytometer (BD Biosciences, NJ).

**Western Blot Analysis**

The protein fraction of cell lysates was resolved by 10%SDS/PAGE before membrane transfer. Primary antibodies were from Santa Cruz Biotechnology (p53), Abcam (p73), Proteintech (MDMX , p21, GAPDH); secondary antibodies conjugated with horseradish peroxidase were from Calbiochem. MG-132 was purchased from Aladdin. Cycloheximide (CHX) was purchased from MeChemExpress.

**RNA-sequencing (RNA-seq) and analysis**

RNA was isolated from WERI-Rb-1 cells after 12 hours of treatment with Nano-MG@PSI (1 μM) or equal volume of medium using the Direct-zol RNA MiniPrep Kit (Zymo Research).

RNA sequencing libraries were constructed using the NEBNext® Ultra RNA Library Prep Kit for Illumina® (NEB England BioLabs). Fragmented and randomly primed 2 × 150 bp paired-end libraries were sequenced using Illumina HiSeq X Ten. Heat maps and Gene Expression Enrichment Analysis were generated using the Qlucore Omics Explorer 3.2. Pathway analysis was performed using Ingenuity Pathway Analysis (IPA) software.

**Animal ethics**

All animal experiments were performed by following Institution Guidelines and were approved by the Laboratory Animal Center of Xi'an Jiaotong University.

**WERI-Rb1 xenograft tumor.**

Four- to five-weeks-old male athymic nude mice were anesthetized by intraperitoneal injection of sodium barbital combined with topical oxybuprocaine hydrochloride eye drops. All nude mice left eyes, chosen as the tumor-bearing models, were dilated pupils with compound tropicamide drops. $50\times10^{\wedge4}$ WERI-Rb-1 cells, resuspended in 3μl PBS, were gently injected into the vitreous cavity of the left eye using Hamilton micro syringe. Tumor growth and weight were observed in subsequent days. On the 3rd day, intravitreal injection of drugs/PBS for model eyes was performed every 4 days for a total of 3 times. The eyes, hearts, livers, spleens, lungs, kidneys, brains were fixed and sliced for further H&E, tunel and immunohistochemical staining.

**Quantification of nanoparticle accumulation and pharmacokinetics using ICP-MS.**

The gold content inside any organ can be measured using ICP-MS. WERI-Rb1 tumors or organs were resected, weighed, and placed in 50 ml Falcon tubes. They were then digested with 2 ml of nitric acid and 0.5 ml of hydrochloric acid at 70–80 °C overnight. The tissues appeared digested and dissolved. The samples were diluted to 50 ml with deionized water and then filtered with 0.22 μm PES filters (Millipore) using a 10 ml syringe. The filtered digest was then processed using ICP-MS and analyzed using a standard curve derived from stock with a known quantity of gold.

**Pancreatic carcinoma-patient-derived xenograft tumor.**

At the time of primary tumor reductive surgery, a specimen was cut into about 5 mm pieces and implanted into the fossa iliaca of NOD/SCID mice aged 4~5 weeks. Tumor length and width were measured with calipers, and tumor volume was calculated using the following equation: tumor volume (V) = length × $width^2/2$. When the tumors reached approximately 50-100 $mm^3$, mice were administrated intraperitoneally at a dosage of 1.5 mg/kg every other day. Xenografts were collected for formalin-fixed-paraffin embedding (FFPE), snap frozen in liquid nitrogen or subsequently implanted into another set of mice using the same procedure. The hearts, livers, spleens, lungs, kidneys, tumors were sliced for further H&E, TUNEL and immunohistochemical staining.

**Immunohistochemical (IHC) staining**

Sections were cut at 5 μm thickness, deparaffinized and rehydrated. Endogenous peroxidase activity was blocked with hydrogen peroxide/methanol, and antigen retrieval was performed in a

pH 9.0 TE (Tris-EDTA) buffer by autoclave for 10 min. The resultant tissue sections were then incubated with primary antibodies against p53, p73, p21, MDMX and Ki67 at 4°C overnight. After incubation with labeled streptavidin-biotin (LSAB) complex for 15 min, the slides were stained and visualized by using the iView DAB detection system (ZSGB-BIO, P.R. China). Each stained section was evaluated by a minimum of 10 randomly selected ×20 high-power fields for further statistical analysis.

**Toxicity studies**

To assess potential toxicities of repeatedly injecting Nano-MG, we monitored body weight of all mice over the course of treatment and measured hematological indexes as well as organ function indexes after 10-day treatment. Control mice were only implanted with xenograft tumor, but did not receive any treatment. Forty-eight hours after the final infusion, mice were anesthetized, and blood was collected for complete blood count (CBC) determinations, including a white blood cell (WBC) count with differential, a red blood cell (RBC) count, haemoglobin and a platelet count. Besides, blood serum was collected, and alanine aminotransferase (ALT), aspartate transaminase (AST), blood urea nitrogen (BUN) and creatinine (CRE) were measured by using quantitative enzyme-linked immunosorbent assay (ELISA) kits according to the manufacturer's instructions. Animals were then euthanized with carbon dioxide to retrieve organs, which were washed with deionized water before fixation in 4% paraformaldehyde. The tissues were processed routinely, and sections were stained with haematoxilin and eosin (H&E).

# 2. Supplemental Figure

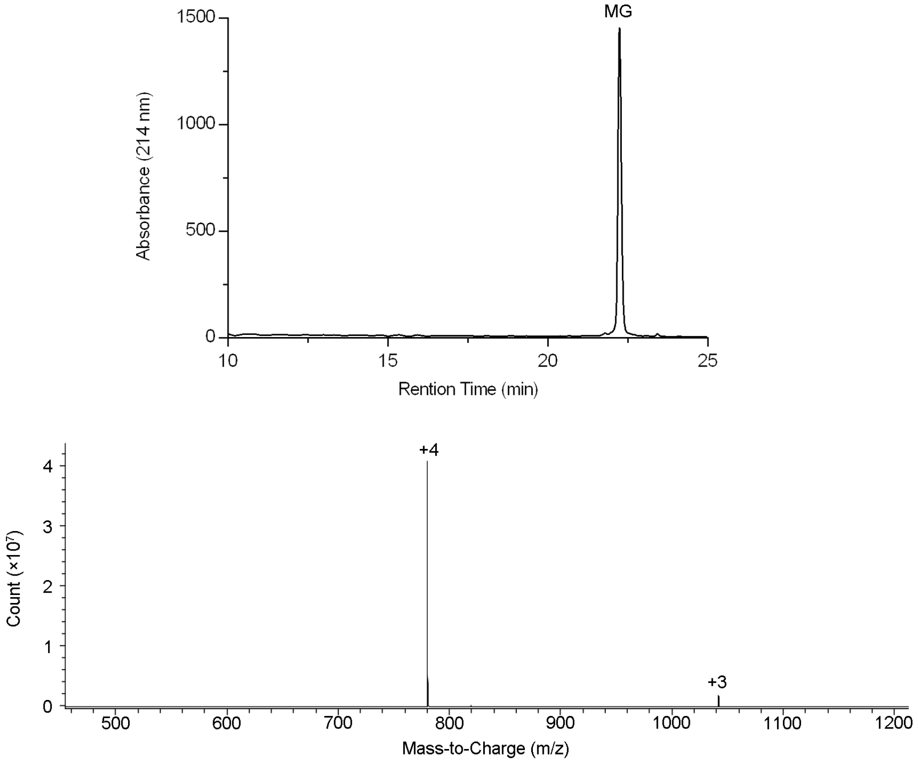


**Figure S1.** Characterization of synthesized MP by HPLC and ESI-MASS.

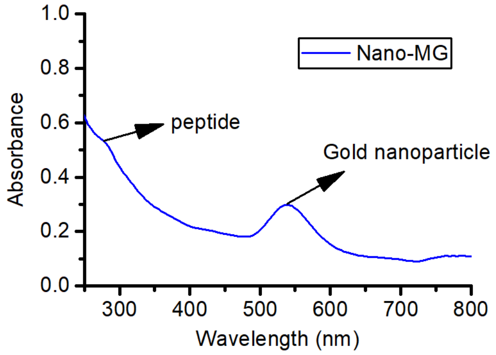


**Figure S2.** Characterization of synthesized Nano-MP by UV-Vis spectrum.

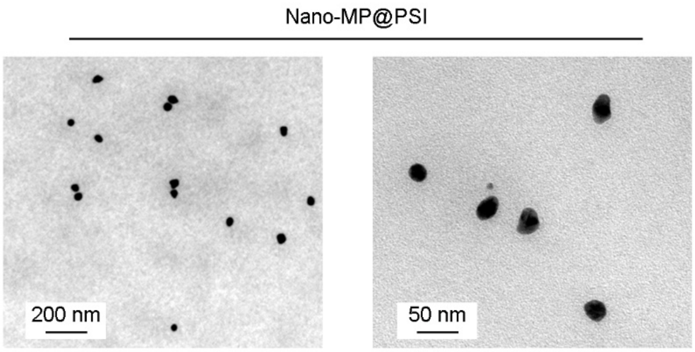


**Figure S3.** TEM images of Nano-MP@PSI.

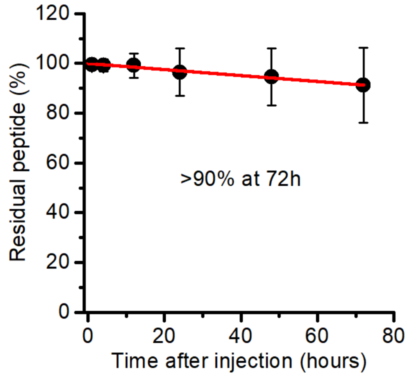


**Figure S4.** Residual MP peptide in Nano-MG@PSI under the incubation of the PBS buffer containing 20% blood serum measured by HPLC.

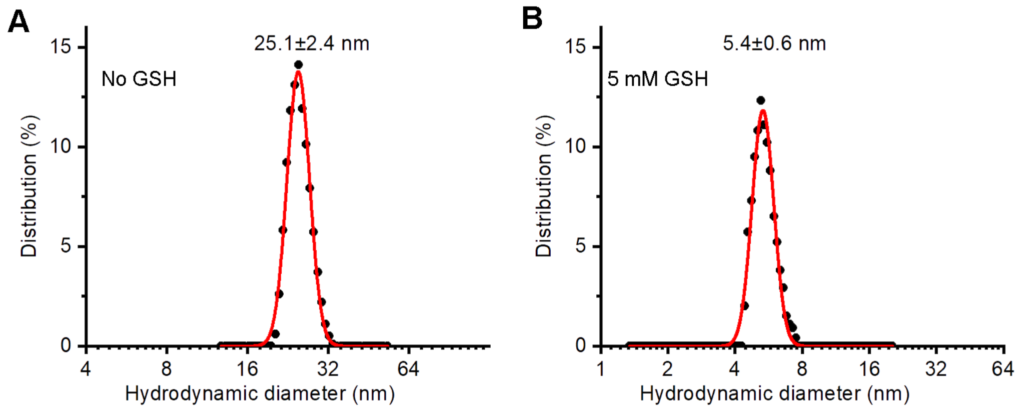


**Figure S5.** Hydrodynamic distributions of Nano-MP before (A) and after (B) 5 mM GSH incubation, which were measured by dynamic light scattering.

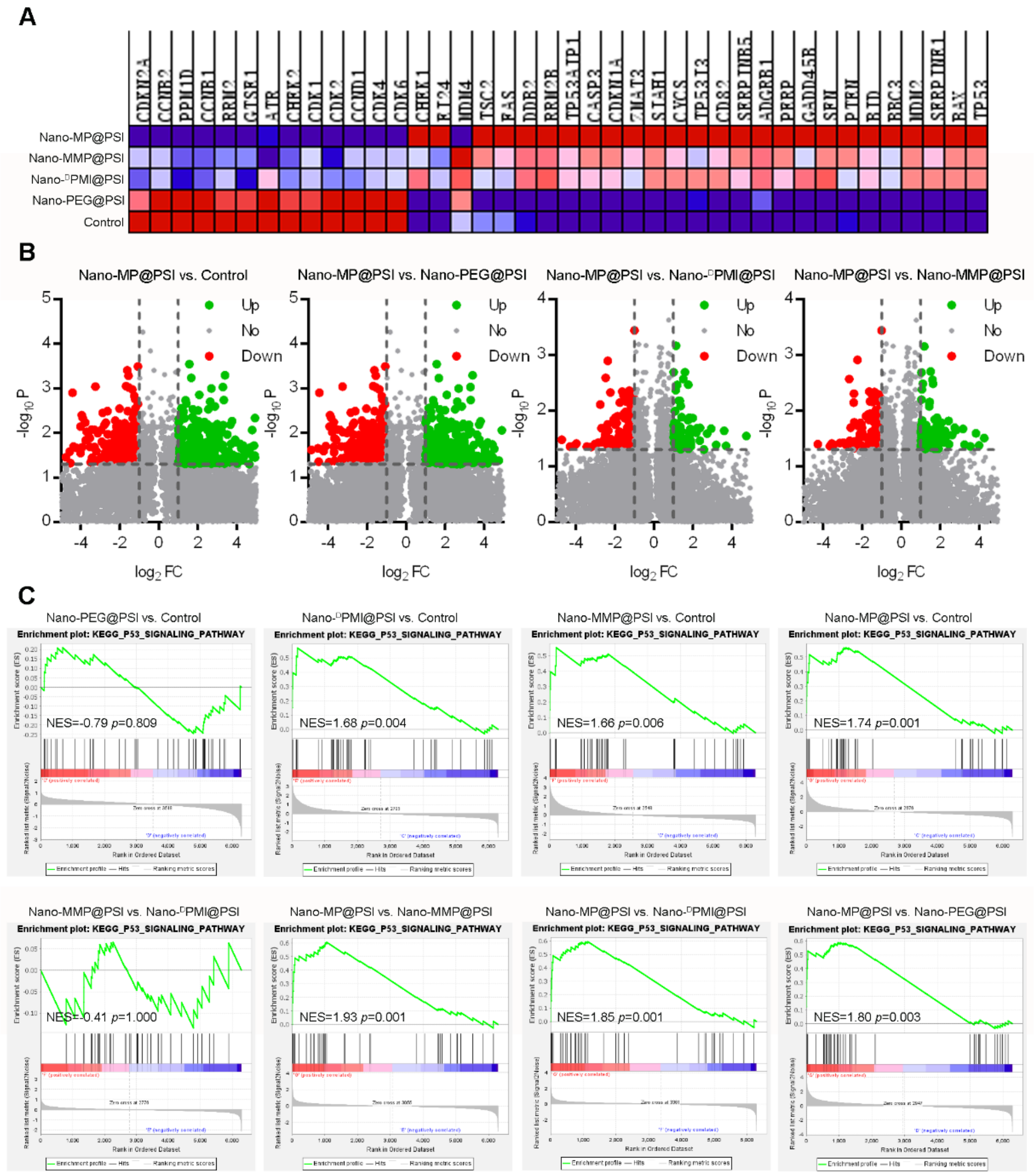


**Figure S6.** Proteomics experiment of WERI-Rb-1 cells treated with Nano-MP@PSI, Nano-$^D$PMI@PSI, Nano-PEG@PSI and Nano-MMP@PSI for 6 h at the dosage of 100 nM. Hierarchical clustering of genes (A), Volcano Plot (B) and gene set enrichment analysis (GSEA, C) and in p53 signaling pathway. N=3 in each group. In Figure S6B, 6290 proteins were found by protein mass spectrometry. 605, 606, 219 and 222 differentially expressed proteins can be found in the comparation of Nano-MP@PSI to Control, Nano-PEG@PSI, Nano-$^D$PMI@PSI and Nano-MMP@PSI, respectively.

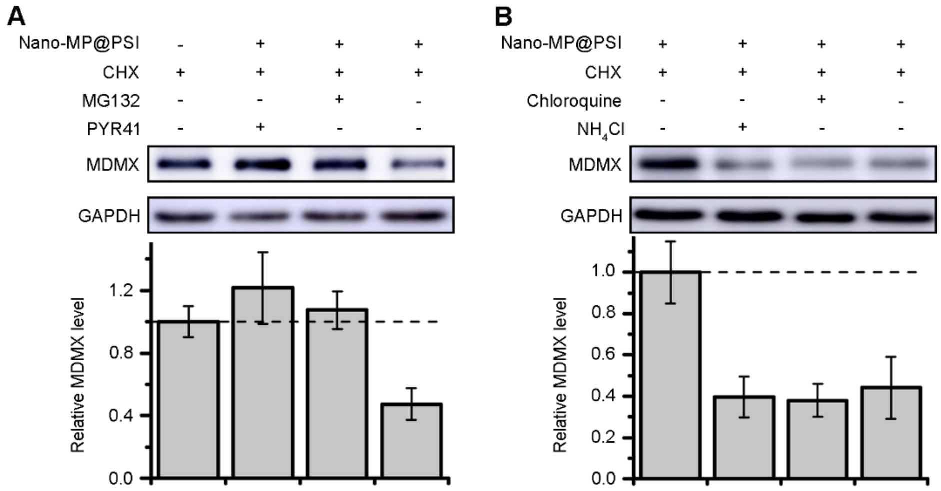


**Figure S7. (A)** The MDMX level responded to Nano-MP@PSI and/or not MG132 (Proteasome inhibitor) or PYR41 (E1 ligase inhibitor). **(B)** The MDMX level responded to Nano-MP@PSI and/or not Chloroquine (lysosome inhibitor) or $NH_4Cl$ (autophagy inhibitor).

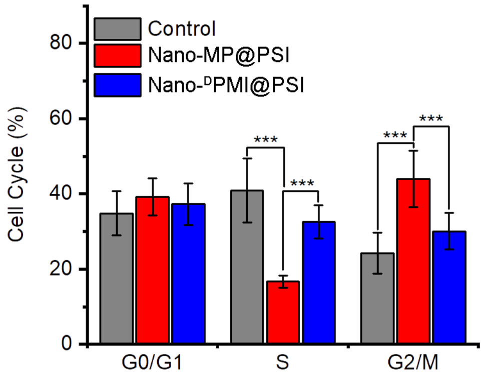


**Figure S8.** Cell cycle distributions of WERI-Rb1 cells with different treatments for 12 h. Data were shown as mean ± s.d. $p$ values were calculated by $t$-test (**, $p$ <0.01; ***, $p$ <0.001).

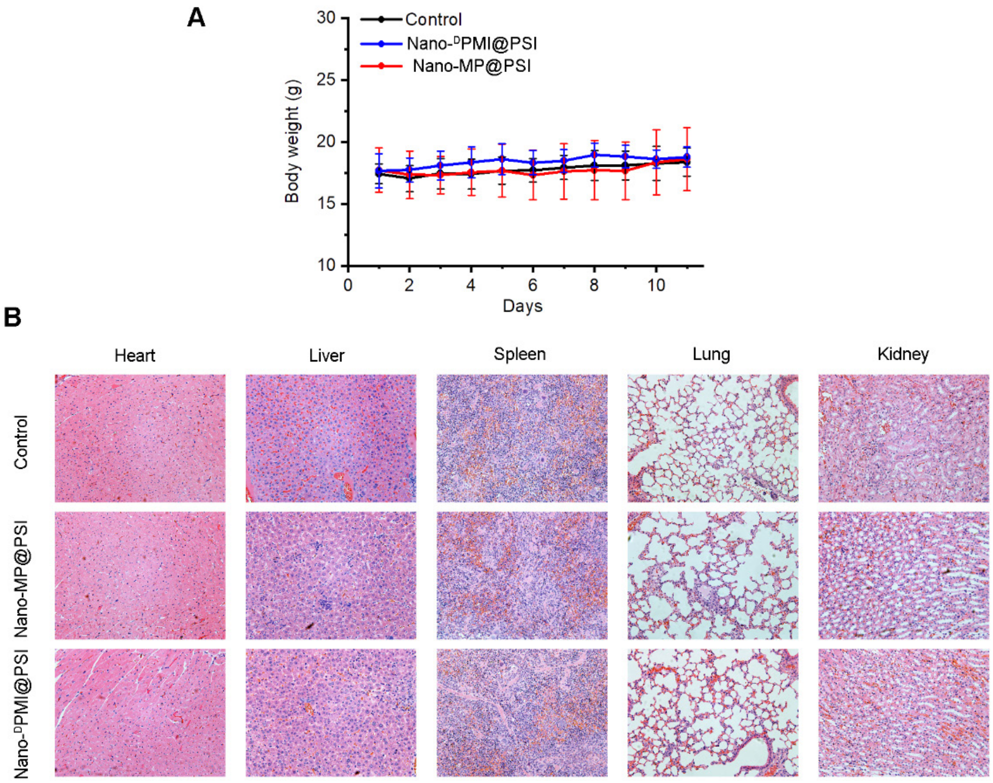


**Figure S9.** A, the weight of mice harboring WERI-Rb1 tumors during the treatments. B, The representative H&E staining of main organ sections in mice with the indicated treatments (X 200).

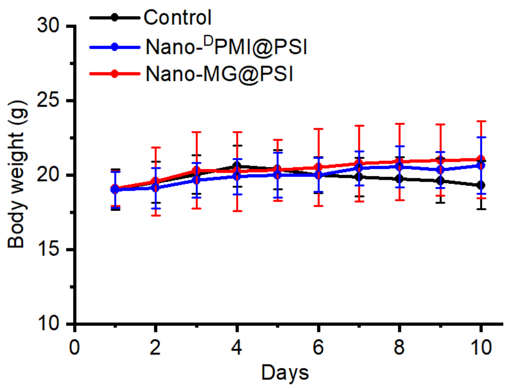


**Figure S10.** The weight of mice harboring PDX tumor of pancreatic carcinoma during the treatments.
